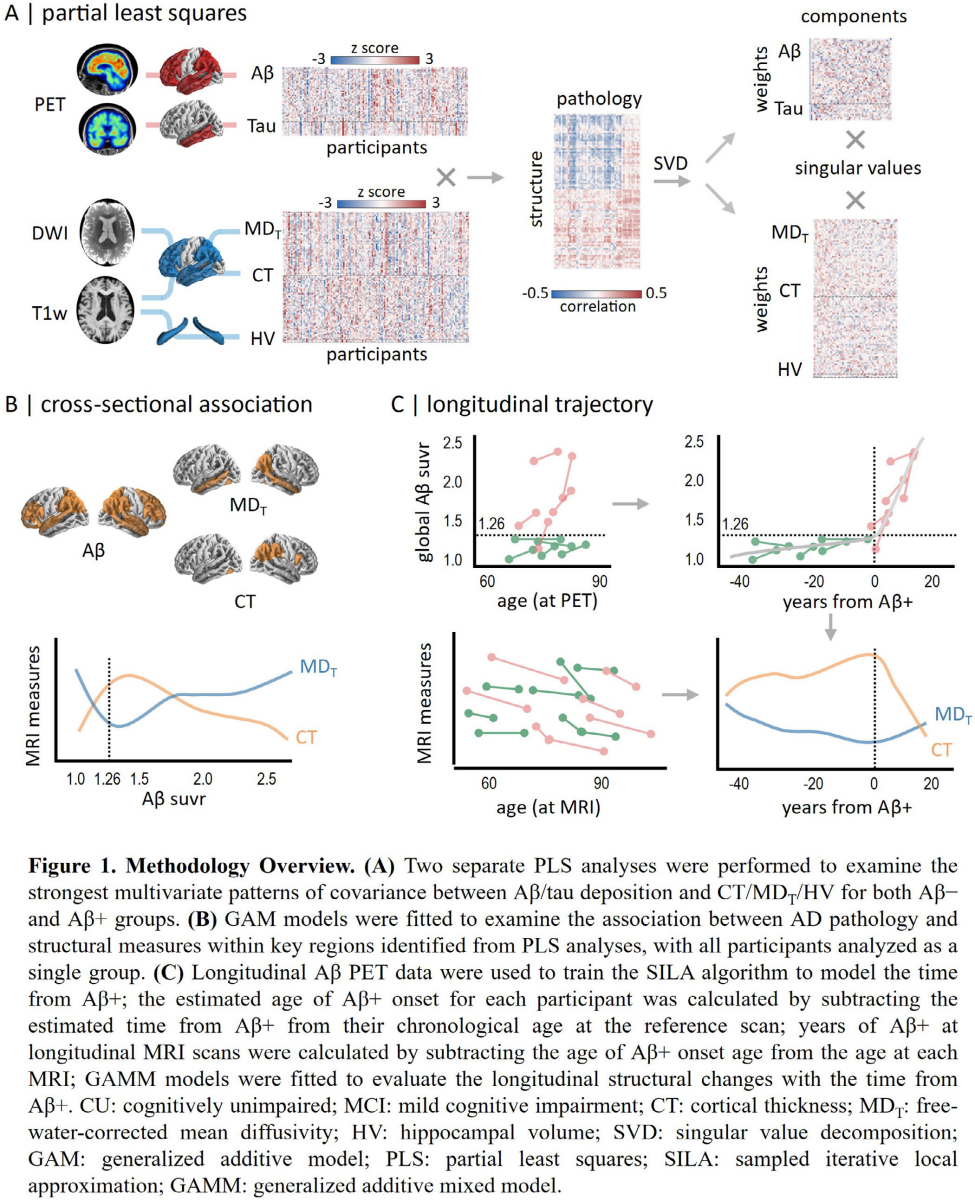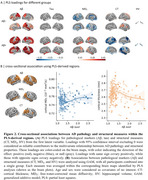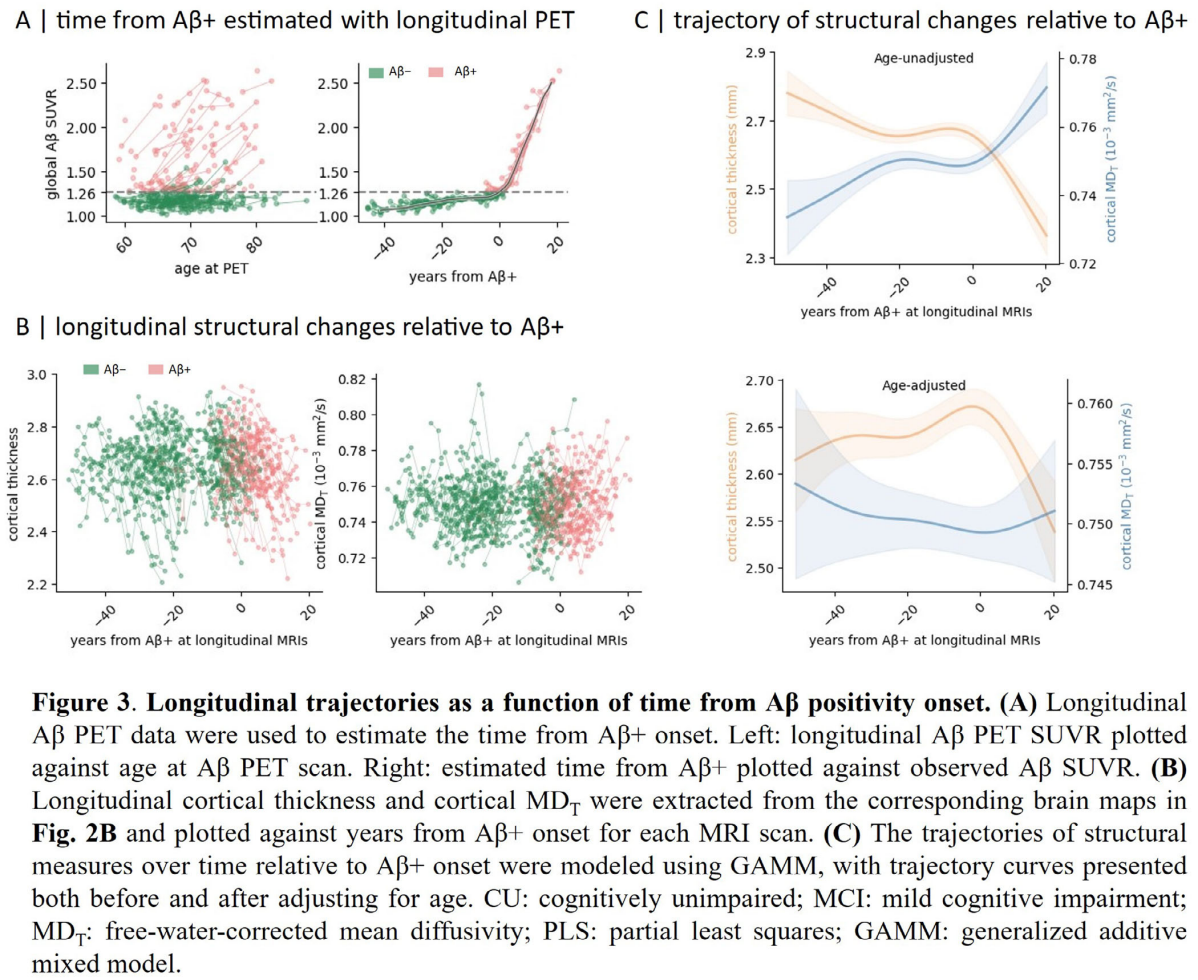# Tracking structural changes in preclinical and prodromal Alzheimer’s disease: insights from amyloid‐beta pathology

**DOI:** 10.1002/alz70862_110868

**Published:** 2025-12-23

**Authors:** Ting Qiu, Zhen‐Qi Liu, Jonathan Gallego Rudolf, Manon Edde, Alex Valcourt Caron, Yuanchao Zhang, Jean‐Paul Soucy, R. Nathan Spreng, Alexa Pichet Binette, Maxime Descoteaux, Sylvia Villeneuve

**Affiliations:** ^1^ Integrated Program in Neurosciences, McGill University, Montréal, QC Canada; ^2^ Centre for Studies on Prevention of Alzheimer's disease (StoP‐AD Centre), Douglas Mental Health Institute, Montreal, QC Canada; ^3^ The Douglas Mental Health Institute, Montreal, QC Canada; ^4^ Douglas Mental Health University Institute, Centre for Studies on the Prevention of Alzheimer's Disease (StoP‐AD), Montréal, QC Canada; ^5^ McGill University, Montreal, QC Canada; ^6^ McConnell Brain Imaging Centre, Montreal Neurological Institute, Montreal, QC Canada; ^7^ Douglas Research Centre, McGill University, Montreal, QC Canada; ^8^ Montreal Neurological Institute, McGill University, Montreal, QC Canada; ^9^ Université de Sherbrooke, Sherbrooke, QC Canada; ^10^ University of Electronic Science and Technology of China, Chengdu, Sichuan China; ^11^ Montreal Neurological Institute, McGill University, Montréal, QC Canada; ^12^ StoP‐AD Centre, Douglas Mental Health Institute Research Centre, Montreal, QC Canada; ^13^ Douglas Mental Health University Institute, Montreal, QC Canada; ^14^ Université de Montréal, Montréal, QC Canada; ^15^ Clinical Memory Research Unit, Department of Clinical Sciences Malmö, Faculty of Medicine, Lund University, Lund Sweden; ^16^ Centre de Recherche de l’Institut Universitaire de Gériatrie de Montréal, Montréal, QC Canada; ^17^ Imeka Solutions Inc, Sherbrooke, QC Canada; ^18^ Department of Psychiatry, McGill University, Montréal, QC Canada; ^19^ Centre for Studies on Prevention of Alzheimer’s Disease (StoP‐AD Centre), Montreal, QC Canada

## Abstract

**Background:**

Amyloid‐beta (Aβ) and tau pathology in Alzheimer’s disease (AD) is commonly associated with disruptions in grey matter integrity, including reduced cortical thickness (CT) and increased cortical mean diffusivity (MD). However, some cross‐sectional studies have also reported an increase in CT during the preclinical stage of the disease. Using over 10 years of longitudinal neuroimaging data from the PREVENT‐AD cohort, we examined the association between AD pathology (Aβ and tau) and brain structure, measured by CT, free‐water corrected MD (MD_T_), and hippocampal volume. We also estimated the longitudinal trajectories of structural changes along the Aβ positivity timeline across the preclinical and prodromal stages of AD.

**Method:**

We performed partial least square analyses (PLS) separately for Aβ negative (Aβ−) and Aβ positive (Aβ+) groups to identify key brain regions that contributed to pathological‐structural associations. We then assessed the cross‐sectional and longitudinal associations between AD pathology and structural measures within the PLS‐identified regions across all participants. Using the sampled iterative local approximation algorithm, we estimated the time from Aβ+ onset and calculated years from Aβ+ for each MRI scan. These estimates allowed us to track the structural changes relative to Aβ positivity (Figure 1).

**Result:**

We found that higher Aβ deposition was associated with decreased MD_T_ and increased CT in the Aβ− group, whereas the Aβ+ group showed opposite associations. Across all participants, associations for MD_T_ followed a U‐shaped pattern, while CT exhibited an inverse U‐shaped relationship with Aβ pathology (Figure 2). These associations were observed in several key AD‐related regions, including the entorhinal cortex, fusiform gyrus, and inferior parietal lobule, and middle temporal regions. Longitudinal analyses revealed similar trajectory patterns along the Aβ+ timeline, with these changes emerging several years before Aβ positivity onset (Figure 3).

**Conclusion:**

Our study suggests that brain structural changes in response to Aβ pathology start decades before symptoms and may follow highly nonlinear trajectories. The initial increases in CT and decreases in MD_T_ might be related to the space taken by Aβ and/or several other biological processes occurring during the preclinical stage of the disease, such as neuroinflammation, astrocytic and microglia activation, or brain swelling.